# Enlarged Perivascular Spaces and Cerebral Small Vessel Disease in Spontaneous Intracerebral Hemorrhage Patients

**DOI:** 10.3389/fneur.2019.00881

**Published:** 2019-08-14

**Authors:** Xin Wang, Hao Feng, Yu Wang, Jian Zhou, Xingquan Zhao

**Affiliations:** ^1^Department of Neurology, Beijing Tiantan Hospital, Capital Medical University, Beijing, China; ^2^Department of Radiology, Beijing Tiantan Hospital, Capital Medical University, Beijing, China

**Keywords:** cerebral small vessel disease, perivascular spaces, intracerebral hemorrhage, risk factors, MRI

## Abstract

**Background:** Cerebral small vessel disease (SVD) is associated with cognitive decline, depression, increased mortality, and disability in stroke patients. MRI-visible perivascular spaces (PVS) are a sensitive neuroimaging marker of SVD. We aimed to explore the risk factors and associations with other SVD markers of PVS in two topographical regions (in the basal ganglia [BG] and centrum semiovale [CS]) in a cohort of spontaneous intracerebral hemorrhage (ICH) patients.

**Method:** We included 306 consecutive patients from a prospective spontaneous ICH cohort. We rated PVS, white matter hyperintensities (WMH), cerebral microbleeds (CMB), and lacunes with validated visual rating scale. We collected clinical information using standardized forms. We predefined severe PVS as score > 2 and examined associations between PVS in both BG and CS regions and clinical and imaging markers of SVD by logistic regression.

**Results:** In the multivariable logistic regression, increasing age (OR = 1.075; 95% CI = 1.038–1.113, *p* < 0.001), high CS PVS degrees (OR = 6.906; 95% CI = 3.024–15.774, *p* < 0.001), extensive periventricular WMH (OR = 2.878; 95% CI = 1.298–6.379, *p* = 0.009), and the presence of CMB (OR = 4.073, 95% CI = 1.869–8.877, *p* < 0.001) were independently associated with BG PVS severity. Alcohol-drinking habit (OR = 2.805; 95% CI = 1.451–5.422, *p* = 0.002), hyperlipidemia history (OR = 3.782; 95% CI = 1.582–8.783, *p* = 0.003), high BG PVS degrees (OR = 6.293; 95% CI = 2.755–14.371, *p* < 0.001) and the presence of strictly lobar CMB (OR = 2.556, 95% CI = 1.285–5.085, *p* = 0.008) were independent predictors of increased CS PVS severity.

**Conclusion:** MRI-visible PVS in BG and CS regions are inter-related and have different risk factors in spontaneous ICH patients. Further studies are needed to explore the mechanism and clinical importance of PVS, with possible implications for cerebrovascular disease prevention and effective treatments.

## Introduction

Spontaneous (non-traumatic) intracerebral hemorrhage (ICH) is a devastating neurological disorder resulting from ruptured blood vessels in the brain (1). ICH is the second most common subtype of stroke (10–15%) and accounts for 2 million strokes worldwide each year (2). However, ICH is the most severe subtype of stroke because of its high fatality case rate and poor functional outcome; the median case fatality is 40.4% at 1 month (1).

Perivascular spaces (PVS), or Virchow–Robin spaces, are fluid-containing spaces that surround the walls of arteries, arterioles, veins, and venules as they course from the subarachnoid space into the brain parenchyma (3, 4). PVS are round or linear delineated structures seen on MRI with intensities close to cerebrospinal fluid (CSF) and <3 mm diameter in cross section (3), and are defined as having a diameter “smaller than 3 mm when imaged perpendicular to the course of the vessel” in the STRIVE guidelines to aid the description of cerebral small vessel disease (SVD) features (5).

PVS may function as fluid circulation and drainage pathways for the efficient exchange of essential nutrients and efficient removal of metabolic waste and cell debris through the central nervous system (6). Though the concept of the “glymphatic system” (7) is controversial (6, 8), this perivascular pathway is important for maintaining brain homeostasis. Increasing visibility of PVS on MRI are associated with increasing age, hypertension (9), stroke, other SVD features such as lacunar stroke and white matter hyperintensities (WMH) (10), systemic inflammation (11), multiple sclerosis (4), cognitive impairment, and dementia (12–14).

In ICH patients, hematomas form rapidly after vessel rupture and lead to a sharp increase in intracranial pressure, which causes primary brain injury. Inflammation begins immediately after the hematoma formation and contributes to secondary brain injury. Immune cells recruitment and infiltration into brain parenchyma is the key step of inflammation initiation and progression (15). PVS are specific sites for immune cell accumulation, reaction, and transmigration into the brain parenchyma (e.g., leukocytes, dendritic cells, T-cells, B-cells, and macrophages (16–18). PVS may provide imaging evidence of vascular and inflammatory changes in the brain after ICH. However, currently there are not many studies that have systematically researched the importance of PVS in spontaneous ICH population (19, 20). Thus, in this study, we aim to investigate the special distribution and severity of PVS in spontaneous ICH patients, and to explore their clinical risk factors and associations with other SVD imaging markers, using structural magnetic resonance imaging (MRI).

## Materials and Methods

### Study Population and Data Collection

For this cross-sectional analysis, we used prospectively collected data from a study of consecutive patients older than 18 years old with spontaneous symptomatic ICH admitted at the Beijing Tiantan Hospital and underwent computed tomography (CT) scans (*N* = 883) from June 2014 to October 2016. Patients were excluded from the study if they did not undergo MRI scans (*N* = 519). Spontaneous symptomatic ICH was confirmed by non-enhanced CT scans showing parenchymal bleeding. Patients were also excluded if they were diagnosed with secondary ICH (*N* = 46), including brain tumor, head trauma, aneurysm, vascular malformation, hemorrhagic infarction, venous infarction, and Moyamoya disease. Patients underwent MRI scans, but those with poor image quality were also excluded (*N* = 12) as further analysis could not be properly performed.

**Figure 1:**
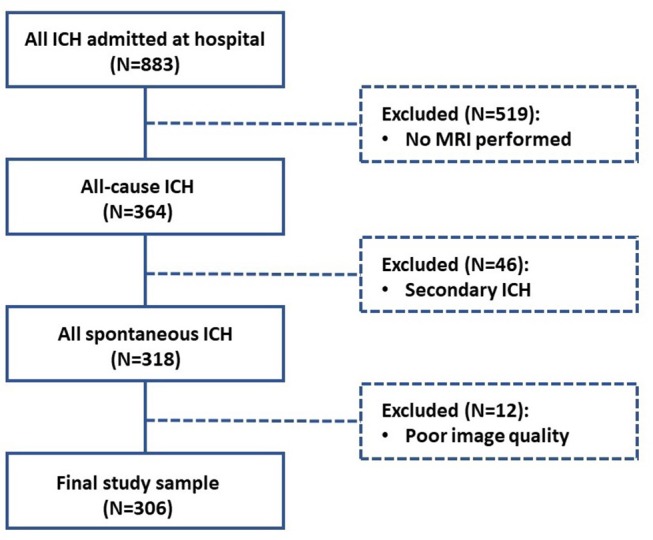
Flow diagram of study enrollment.

We finally included 306 patients diagnosed as spontaneous ICH (Figure 1). All patients participating in this study provided written informed consent. The Beijing Tiantan Hospital Ethics Committee approved this study. Trained stroke physicians collected detailed patient information by using standard questionnaires at presentation. Age, gender, and clinical history (hypertension, diabetes, hypercholesterolemia, cerebral infarction, intracerebral hemorrhage (ICH), subarachnoid hemorrhage, transient ischemic attack (TIA), atrial fibrillation, myocardial infarction, heart failure and other heart disease, and peripheral vascular disease) were systematically recorded for each patient. Cigarette smoking was classified as never, previous, or current smokers, and alcohol drinking was classified as current drinker or non-drinker. Patients underwent CT, MRI, carotid imaging, and other investigations as required for following ICH treatment.

### Brain MRI Acquisition

For all participants, MRI were performed within 6 days of admission. Images were obtained with two 3T MRI scanners, GE Discovery 750 scanner (GE Healthcare, Milwaukee, Wis) and Siemens verio scanner (Siemens, Erlangen, Germany). The MRI ICH protocols are similar in two MRI scanners, including the following sequences: whole brain T1 weighted image (T1W; repetition time/echo time 1,900/9.4 ms; 5 mm slice thickness, 6 mm interslice gap), T2-weighted image (T2W; repetition time/echo time 6,000/97 ms, 5 mm slice thickness, 6 mm interslice gap), and fluid-attenuated inversion recovery (FLAIR; repetition time/echo time 7,800/91 ms, inversion time 2,200 ms, 5 mm slice thickness, 6 mm interslice gap) and susceptibility-weighted imaging (SWI; repetition time/echo time 28/20 ms, flip angle 15°, 1.6 mm slice thickness, 0.3 mm interslice gap).

### Neuroimaging Analysis

All neuroimaging analysis were performed and recorded according to STandards for ReportIng Vascular changes on nEuroimaging (STRIVE) guidelines (5) by trained image analysts blinded to patients' clinical characteristics. PVS were defined as ≤ 3 mm round or linear CSF isointensity lesions and were rated on axial T2W imaging with a validated visual rating scale (10, 21): rated from 0 (none), 1 (1–10), 2 (11–20), 3 (21–40), and 4 (>40) in both the basal ganglia (BG) and centrum semiovale (CS) regions. For both BG and CS regions, after going through all relevant slices for the anatomical area being assessed, we reviewed at least 3 adjacent slices with extensive PVS. One slice with the highest number of PVS was selected and the rating score on this selected slice was recorded. In cases with extensive WMH in the CS region, an estimate was made of the closest PVS rating category, using the appearance of non-involved white matter and cortical gray matter. When background brain parenchyma is asymmetric due to large lobar or deep ICH, PVS were rated in the contralateral hemisphere, and an estimate was made of the closest PVS rating category ipsilateral to the large lesion.

Lacunes were distinguished from PVS by larger size (3~15 mm) on axial FLAIR imaging and defined as a subcortical round or ovoid fluid-filled cavity with a similar signal intensity as CSF (5). WMH are hyperintense lesions on FLAIR and T2W imaging and appear as hypointense on T1W imaging (5). We rated periventricular and deep WMH as 0–3 with the Fazekas scale (22) on FLAIR or T2W imaging. Periventricular WMH was rated from 0 (none), 1 (pencil-thin lining), 2 (smooth halo), and 3 (irregular signal extending to the deep white matter). Deep WMH was rated from 0 (none), 1 (punctate foci), 2 (beginning confluence), and 3 (large confluent areas). Microbleeds were defined as small (generally 2–5 mm in diameter, but up to 10 mm), rounded, or circular areas of signal void on susceptibility sensitive sequences. According to the Microbleed Anatomical Rating Scale (23), microbleeds were classified into deep, lobar, and infratentorial categories. Deep regions included the basal ganglia, thalamus, internal capsule, external capsule, corpus callosum, and deep and periventricular white matter; lobar regions included cortical and subcortical regions; infratentorial regions included the brainstem and cerebellum. We rated the number of microbleeds according to locations separately on SWI imaging and summed them up as total microbleeds. Microbleed mimics were carefully excluded using CT scans or T2W and FLAIR imaging. Basal ganglia calcification can mimic microbleeds and was excluded using CT scans. Mimics of sulcal vessel seen in cross section and partial volume artifact from adjacent bony structures were excluded by careful inspection of adjacent slices and reference to available T2W and FLAIR images.

All MRIs were assessed blinded to clinical information and the other rater's ratings. The interrater Cohen's kappa value for BG PVS was 0.91 and CS PVS was 0.87, for lacunes was 0.89, for WMH was 0.86–0.89, and for microbleeds was 0.83–0.90.

### Statistical Analysis

We classified the PVS burden as high (score 3 and 4) or low (score 0–2) as suggested in previous studies (9, 20). We defined the extensive WMH as irregular periventricular WMH extending into the deep white matter (Fazekas score 3) or early confluent/confluent deep WMH (Fazekas score 2 or 3) as suggested previously (24, 25). Clinical and imaging characteristics of patients with high PVS degree vs. low PVS degree were compared in univariate analyses with the 2-sample *t*-test, Pearson's chi-squared test and Fisher exact test when appropriate. We subsequently evaluated independent predictors of high PVS degree in both BG and CS regions by logistic regression analyses based on univariable analyses results. Logistic regression models were run with a stepwise, forward-elimination method to generate a minimal adjusted model. All tests of significance were 2 tailed. Significance level was set at 0.05 for all analyses. SPSS software (SPSS for Windows, version 22.0, IBM-SPSS, Chicago, IL) was used for all analyses.

## Results

Our final cohort included 306 patients with spontaneous ICH. The mean age was 56.00 (±13.27) years old, ranging from 18 to 86 years old; the proportion of males was 71.57% (219 patients); and more than half of the cohort were current or previous smokers (50.33%) and have the alcohol-drinking habit (50.33%, Table 1). In this cohort, 73.20% of patients had been diagnosed as hypertension previously, 13.73% had diabetes mellitus, and 11.11% have hypercholesterolemia problems. No patients had the history of TIA, myocardial infarction, heart failure, or peripheral vascular disease. Nearly 15% of patients had prior cerebral infarction, and seven patients had prior cerebral hemorrhage (seven ICH and one of them also had subarachnoid hemorrhage). Fifteen patients had heart diseases (4.90%); including two patients who had atrial fibrillation (0.65%, Table 1).

**Table 1:** Baseline characteristics of all spontaneous ICH patients (*n* = 306).

**Characteristics**	
**DEMOGRAPHICS**	
Mean age in years (SD)	56.00 (13.27)
Male gender no. (%)	219 (71.57%)
**VASCULAR RISK FACTORS**	
Ever-smokers no. (%)	154 (50.33%)
Alcohol drinking no. (%)	154 (50.33%)
Hypertension no. (%)	224 (73.20%)
Diabetes mellitus no. (%)	42 (13.73%)
Hyperlipidemia no. (%)	34 (11.11%)
Prior cerebral infarction no. (%)	44 (14.38%)
Prior intracerebral hemorrhage no. (%)	7 (2.29%)
Prior subarachnoid hemorrhage no. (%)	1 (0.33%)
Artial fibrillation no. (%)	2 (0.65%)
Other heart disease no. (%)	13 (4.25%)
Mean baseline SBP (SD)	163.58 (24.14)
Mean baseline DBP (SD)	95.38 (17.53)
**SVD MARKERS**	
Basal ganglia PVS, median (IQR)	2 (1–2)
Rating 0, no. (%)	6 (1.96%)
Rating 1, no. (%)	142 (46.41%)
Rating 2, no. (%)	107 (34.97%)
Rating 3, no. (%)	39 (12.75%)
Rating 4, no. (%)	12 (3.92%)
Centrum semiovale PVS, median (IQR)	2 (1–2)
Rating 0, no. (%)	11 (3.59%)
Rating 1, no. (%)	141 (46.08%)
Rating 2, no. (%)	95 (31.05%)
Rating 3, no. (%)	49 (16.01%)
Rating 4, no. (%)	10 (3.27%)
Periventricular WMH Fazekas score, median (IQR)	2 (1–2)
Grade 0, no. (%)	14 (4.58%)
Grade 1, no. (%)	104 (33.99%)
Grade 2, no. (%)	126 (41.18%)
Grade 3, no. (%)	62 (20.26%)
Deep WMH Fazekas score, median (IQR)	1 (1–2)
Grade 0, no. (%)	39 (12.75%)
Grade 1, no. (%)	116 (37.91%)
Grade 2, no. (%)	114 (37.25%)
Grade 3, no. (%)	37 (12.09%)
Microbleeds, median (IQR)	1 (0–3)
Presence of microbleeds, no. (%)	156 (50.98%)
1 microbleed, no. (%)	58 (18.95%)
2–4 microbleeds, no. (%)	56 (18.30%)
>5 microbleeds, no. (%)	42 (13.73%)
Strictly deep microbleeds, no. (%)	64 (20.92%)
Strictly lobar microbleeds, no. (%)	13 (4.25%)
Lacune, median (IQR)	0 (0–2)
Presence of lacunes, *n* (%)	133 (43.46%)

*DBP, diastolic blood pressure; IQR, interquartile range; PVS, enlarged perivascular spaces; SBP, systolic blood pressure; SD, standard deviation; SVD, small vessel disease; WMH, white matter hyperintensities. Ever smokers indicate current and previous smokers. Other heart disease does not include myocardial infarction and heart failure*.

Table 1 also presents radiological characteristics of all ICH patients. According to the definitions and SVD scales used for visual rating, BG PVS (98.04%), CS PVS (96.41%), and periventricular WMH (95.42%) were the most prevalent, followed by deep WMH (87.25%), presence of microbleeds (50.98%) and lacunes (43.46%). The median BG PVS is 2 (interquartile range: IQR = 1–2) and the median CS PVS is also 2 (IQR = 1–2).

### Predictors of Increased BG PVS Severity in Spontaneous ICH Patients

Demographic, vascular, and radiological characteristics of patients with high and low BG PVS degree are compared in Table 2. In univariable analyses, high BG PVS degree was significantly associated with increasing age (*p* < 0.001), hyperlipidemia history (*p* = 0.034), and baseline systolic blood pressure (*p* = 0.049). High BG PVS degree was also significantly associated high degree of PVS in CS region (*p* < 0.001), extensive WMH in both periventricular and deep regions (both *p* < 0.001) and presence of lacunes (*p* = 0.002). High degrees of BG PVS were associated with the presence of CMB (*p* < 0.001), but not with strictly deep CMB (*p* = 0.379) or strictly lobar CMB (*p* = 1.000).

**Table 2:** Comparison of characteristics between patients with high and low degree of PVS in the BG region.

**Characteristic**	**High BG PVS degree**	**Low BG PVS degree**	***p-*value**
Demographics	(BG PVS > 2, *n* = 51)	(BG PVS ≤ 2, *n* = 205)	
Mean age in years (SD)	64.53 (10.73)	54.29 (13.08)	*p* < 0.001
Male gender no. (%)	39 (76.47%)	180 (70.59%)	*p* = 0.395
**VASCULAR RISK FACTORS**			
Ever-smokers no. (%)	27 (52.94%)	127 (49.80%)	*p* = 0.682
Alcohol drinking no. (%)	29 (56.86%)	125 (49.02%)	*p* = 0.306
Hypertension no. (%)	38 (74.51%)	186 (72.94%)	*p* = 0.817
Diabetes mellitus no. (%)	6 (11.76%)	36 (14.12%)	*p* = 0.656
Hyperlipidemia no. (%)	10 (19.61%)	24 (9.41%)	*p* = 0.034
Prior cerebral infarction no. (%)	10 (19.61%)	34 (13.33%)	*p* = 0.244
Prior intracerebral hemorrhage no. (%)	1 (1.96%)	6 (2.35%)	*p* = 1.000
Prior subarachnoid hemorrhage no. (%)	1 (1.96%)	0 (0.00%)	*p* = 0.167
Artial fibrillation no. (%)	1 (1.96%)	1 (0.39%)	*p* = 3.060
Other heart disease no. (%)	2 (3.92%)	11 (4.31%)	*p* = 1.000
Mean baseline SBP (SD)	157.51 (20.41)	164.75 (24.67)	*p* = 0.049
Mean baseline DBP (SD)	91.63 (15.65)	96.13 (17.82)	*p* = 0.094
**SVD MARKERS**			
CS PVS median (IQR)	2 (1–3)	1 (1–2)	/
High degree CS PVS, no. (%)	20 (39.22%)	39 (15.29%)	*p* < 0.001
Periventricular WMH Fazekas score, median (IQR)	2 (2–3)	2 (1–2)	/
Extensive periventricular WMH (score = 3), no. (%)	24 (47.06%)	38 (14.90%)	*p* < 0.001
Deep WMH Fazekas score, median (IQR)	2 (2–3)	2 (1–2)	/
Extensive deep WMH (score ≥ 2), no. (%)	24 (47.06%)	38 (14.90%)	*p* < 0.001
Microbleeds, median (IQR)	2 (1–7)	0 (0–2)	/
Presence of microbleeds, no. (%)	39 (76.47%)	117 (45.88%)	*p* < 0.001
Strictly deep microbleeds, no. (%)	13 (25.49%)	51 (20.00%)	*p* = 0.379
Strictly lobar microbleeds, no. (%)	2 (3.92%)	11 (4.31%)	*p* = 1.000
Lacunes, median (IQR)	1 (0–3)	0 (0–1)	/
Presence of lacunes, no. (%)	32 (62.75%)	101 (39.61%)	*p* = 0.002

*BG, basal ganglia; CS, centrum semiovale; DBP, diastolic blood pressure; IQR, interquartile range; PVS, enlarged perivascular spaces; SBP, systolic blood pressure; SD, standard deviation; SVD, small vessel disease; WMH, white matter hyperintensities. Ever smokers indicate current and previous smokers. Other heart disease does not include myocardial infarction and heart failure. Extensive periventricular WMH indicates periventricular WMH Fazekas score 3 and extensive deep WMH indicates deep Fazekas scores 2–3*.

In multivariable logistic regression analysis (Table 4), increasing age (OR = 1.075; 95% CI = 1.038–1.113, *p* < 0.001) was an independent predictor of increased BG PVS severity, after adjusting for gender and hypertension. The association between high degrees of BG PVS and high CS PVS degrees (OR = 6.906; 95% CI = 3.024–15.774, *p* < 0.001), extensive WMH in periventricular region (OR = 2.878; 95% CI = 1.298–6.379, *p* = 0.009), the presence of CMB (OR = 4.073, 95% CI = 1.869–8.877, *p* < 0.001) remained significant after adjustment for age, gender, and hypertension.

### Predictors of Increased CS PVS Severity in Spontaneous ICH Patients

Demographic, vascular, and radiological characteristics of patients with high and low CS PVS degree are compared in Table 3. In univariable analyses, severe CS PVS were associated with the male gender (*p* = 0.030), an alcohol-drinking habit (*p* = 0.007), and hyperlipidemia history (*p* = 0.001). High degrees of CS PVS were also associated with high degrees of BG PVS (*p* < 0.001) and the presence of strictly lobar CMB (*p* = 0.012).

**Table 3:** Comparison of characteristics between patients with high and low degree of PVS in the CS region.

**Characteristic**	**High CS PVS degree**	**Low CS PVS degree**	***p-*value**
Demographics	(CS PVS > 2, *n* = 59)	(CS PVS ≤ 2, *n* = 247)	
Mean age in years (SD)	54.58 (12.12)	56.34 (13.59)	*p* = 0.361
Male gender no. (%)	49 (83.05%)	170 (68.83%)	*p* = 0.030
**VASCULAR RISK FACTORS**			
Ever-smokers no. (%)	28 (47.46%)	126 (51.01%)	*p* = 0.624
Alcohol drinking no. (%)	39 (66.10%)	115 (46.56%)	*p* = 0.007
Hypertension no. (%)	42 (71.19%)	182 (73.68%)	*p* = 0.697
Diabetes mellitus no. (%)	11 (18.64%)	31 (12.55%)	*p* = 0.222
Hyperlipidemia no. (%)	14 (23.73%)	20 (8.10%)	*p* = 0.001
Prior cerebral infarction no. (%)	9 (15.25%)	35 (14.17%)	*p* = 0.831
Prior intracerebral hemorrhage no. (%)	2 (3.39%)	5 (2.02%)	*p* = 0.528
Prior subarachnoid hemorrhage no. (%)	1 (1.69%)	0 (0.00%)	*p* = 0.93
Artial fibrillation no. (%)	1 (1.69%)	1 (0.40%)	*p* = 0.349
Other heart disease no. (%)	3 (5.08%)	10 (4.05%)	*p* = 0.723
Mean baseline SBP (SD)	164.05 (22.90)	163.46 (24.47)	*p* = 0.867
Mean baseline DBP (SD)	98.39 (19.59)	94.66 (16.96)	*p* = 0.143
**SVD MARKERS**			
BG PVS median (IQR)	2 (1–3)	1 (1–2)	/
High degree BG PVS, no. (%)	20 (33.90%)	31 (12.55%)	*p* < 0.001
Periventricular WMH Fazekas score, median (IQR)	2 (1–2)	2 (1–2)	/
Extensive periventricular WMH (score = 3), no. (%)	9 (15.25%)	53 (21.46%)	*p* = 0.287
Deep WMH Fazekas score, median (IQR)	2 (1–2)	1 (1–2)	/
Extensive deep WMH (score ≥ 2), no. (%)	34 (57.63%)	117 (47.37%)	*p* = 0.157
Microbleeds, median (IQR)	1 (0–2)	1 (0–3)	/
Presence of microbleeds, no. (%)	30 (50.85%)	126 (51.01%)	*p* = 0.982
Strictly deep microbleeds, no. (%)	13 (22.03%)	51 (20.65%)	*p* = 0.814
Strictly lobar microbleeds, no. (%)	6 (10.17%)	7 (2.83%)	*p* = 0.012
Lacunes, median (IQR)	1 (1–2)	0 (0–2)	/
Presence of lacunes, no. (%)	30 (50.85%)	103 (41.70%)	*p* = 0.203

*BG, basal ganglia; CS, centrum semiovale; DBP, diastolic blood pressure; IQR, interquartile range; PVS, enlarged perivascular spaces; SBP, systolic blood pressure; SD, standard deviation; SVD, small vessel disease; WMH, white matter hyperintensities. Ever smokers indicate current and previous smokers. Other heart disease does not include myocardial infarction and heart failure. Extensive periventricular WMH indicates periventricular WMH Fazekas score 3 and extensive deep WMH indicates deep Fazekas scores 2–3*.

In multivariable logistic regression analysis (Table 4), an alcohol-drinking habit (OR = 2.805; 95% CI = 1.451–5.422, *p* = 0.002) and hyperlipidemia history (OR = 3.782; 95% CI = 1.582–8.783, *p* = 0.003) were independent predictors of increased CS PVS severity, after adjusting for age, gender, and hypertension. The association between high CS PVS degrees and high BG PVS degrees (OR = 6.293; 95% CI = 2.755–14.371, *p* < 0.001) and the presence of strictly lobar CMB (OR = 2.556, 95% CI = 1.285–5.085, *p* = 0.008) remained significant.

**Table 4:** Univariable and multivariable associations for high degree of BG PVS and high degree of CS PVS.

	**Unadjusted OR (95% CI), *p-*value**	**Adjusted OR (95% CI), *p-*value**
**HIGH DEGREE OF BG PVS**		
Age	1.070 (1.041–1.100), *p* < 0.001	1.075 (1.038–1.113), *p* < 0.001
High CS PVS degree	3.573 (1.851–6.896), *p* < 0.001	6.906 (3.024–15.774), *p* < 0.001
Extensive periventricular WMH	5.076 (2.653–9.713), *p* < 0.001	2.878 (1.298–6.379), *p* = 0.009
Presence of microbleeds	3.833 (1.918–7.660), *p* < 0.001	4.073 (1.869–8.877), *p* < 0.001
**HIGH DEGREE OF CS PVS**		
Alcohol drinking	2.792 (1.455–5.358), *p* = 0.002	2.805 (1.451–5.422), *p* = 0.002
Hyperlipidemia	3.265 (1.424–7.485), *p* = 0.005	3.728 (1.582–8.783), *p* = 0.003
High BG PVS degree	4.299 (2.030–9.102), *p* < 0.001	6.293 (2.755–14.371), *p* < 0.001
Strictly lobe microbleeds	2.422 (1.229–4.773), *p* = 0.011	2.556 (1.285–5.085), *p* = 0.008

*BG, basal ganglia; CI, confidence interval; CS, centrum semiovale; OR, odds ratio; PVS, enlarged perivascular spaces; WMH, white matter hyperintensities. Age effects are presented per year. Multivariable logistic regression model adjusted for age, gender, and hypertension*.

## Discussion

In this consecutive cohort of spontaneous ICH patients, we find that MR-visible BG PVS and CS PVS are closely co-associated. BG PVS severity is independently associated with increasing age, extensive periventricular WMH and presence of MB. CS PVS severity is associated with alcohol-drinking habit, hypercholesterolemia history, and the presence of strictly lobar MB.

Our results are in agreement with previous studies that BG and CS PVS are closely co-associated. There was a significant relationship between BG PVS and CS PVS severity in healthy elderly subjects (26) or in patients with acute ischemic stroke (IS) or TIA (10, 13, 27). However, a previous study described no statistically significant association between BG PVS and CS PVS severity in spontaneous ICH patients (19). PVS functions as fluid circulation and drainage pathways for efficient exchange of essential nutrients and efficient removal of metabolic waste and cell debris through the central nervous system, and form part of the glymphatic system (28). Both visibility of BG PVS and CS PVS on MRI could be considered as a marker of dysfunctional perivascular flow, impaired nutrient change and waste flushing (29). In parallel, the arterial wall thickening and stiffening occurring with aging or hypertension impairs normal perivascular fluid flushing, facilitating dilation of PVS (30, 31). The dysfunction of the blood–brain barrier might contribute to the dilation of PVS in both regions (29). A variety of study groups show advancing age is the strongest independent risk factor for PVS in both BG and CS regions in healthy elderly subjects (9), in IS patients (10), and in patients with CAA-ICH patients (19). Our results are in line with these previous studies that we find BG PVS is associated with increasing age in this cohort of spontaneous ICH patients, but we did not find the same association between increasing age and CS PVS severity. This difference may relate to different brain integrity status, underlying pathophysiological processes and genotypes among patients (26, 32).

We identified a positive association between BG PVS severity and extensive periventricular WMH in ICH patients after adjusting for age, gender and hypertension. Arba et al. (13) found that in patients with IS or TIA, BG PVS severity was associated with WMH severity in both periventricular and deep regions. In another group of IS and TIA patients, Hurford et al. (33) found severe WMH was independently associated with BG PVS severity, however, they did not specify the WMH regions and used a different WMH visual rating scale. In a large memory clinic cohort, both high degrees of BG PVS and CS PVS were associated with moderate-to-severe WMH after adjustment and they did not specify the periventricular and deep WMH either (34). In the Kashima Scan Study, BG PVS severity was associated with severe WMH but not periventricular WMH (35) in neurologically healthy adults. In a recent published systematic review and meta-analysis about the association between PVS and neuroimaging features, the association between PVS and WMH was not significant in the meta-analysis though the direction is positive (36) and the PVS-WMH associations may reflect differences in population characteristics or shared co-associations. Increased WMH burden is associated with blood–brain barrier dysfunction in patients with SVD (37–39). The blood–brain barrier dysfunction might facilitate dilation of PVS, endothelial dysfunction, and impair further nutrient transport. Endothelial dysfunction blocked oligodendrocyte precursor cell maturation, thereby causing direct damage to myelin and myelin repairment (40, 41). The blood–brain barrier dysfunction might contribute to both BG PVS severity and extensive periventricular WMH (29).

Our study suggested the association between PVS and CMB varied by regions. We found the BG PVS severity was associated with the presence of CMB (at least one microbleed appearing at any region) and the CS PVS severity as associated with the presence of strictly lobar CMB. Our study agrees with previous studies that CS PVS severity was associated with strictly lobar CMB in neurologically healthy adults (35) and increased lobar CMB count was an independent predictor of high CS PVS degrees in a memory clinic cohort (42). However, in this study we did not find the association between the BG PVS severity and strictly deep CMB observed in previous studies including in ICH patients (19) and in a large memory clinic cohort (34). Currently there were insufficient data to compare PVS and CMB by locations (36), and the association between PVS and CMB in different locations merits attention in future studies.

We identified that current alcohol-drinking status is significantly associated with CS PVS severity in ICH patients after adjusting for age, gender, and hypertension. Other groups have studied alcohol intake and the risk of ICH and SVD. Heavy alcohol intake (more than 300 g alcohol per week) was associated with the spontaneous ICH occurrence at a younger age (43) and deep CMB incident in a population-based study (44). Current alcohol-drinking status was associated with WMH volume in acute ischemic stroke patients (45) and silent brain infarction in community-dwelling elderly people (46). In this study, we recorded the patient current alcohol drinking status instead of alcohol consumed in grams, in accordance with the suggestion that consuming zero standard drinks, daily, minimizes health risks, from the UK's chief medical officer guidelines (47) and the global burden of disease 2016 (48).

We found a positive association between hypercholesterolemia history and severe CS PVS degree in ICH patients; however, a previous study found higher blood total cholesterol levels was inversely associated with PVS severity in CS region in healthy elderly subjects (9). Interestingly, previous studies suggested hypercholesterolemia might play a protective role in SVD features such as WMH and infarction: IS patients and control participants with hypercholesterolemia history had less severe WMH (49); IS patients with high triglyceride levels had less WMH severity (50) and higher blood total cholesterol levels were significantly associated with a lower risk of WMH and lacunar infarction in participants aged 40 and over (51). The link between PVS severity and hypercholesterolemia history or blood cholesterol levels deserves more attention and further investigations.

Major strengths of our study include a large consecutive cohort of spontaneous ICH patients which were reviewed by ICH panel experts; furthermore, we used a highly standardized SVD visual methodology and were blinded to patients' clinical information and risk factor profiles to avoid rating bias. Potential limitations are in the study design. This cohort only consists of patients with spontaneous ICH with good quality MRI scans (after actively excluding patients diagnosed with secondary ICH), and a potential limitation is the lack of a control group with age-matched healthy patients which might limit the generalizability of the results. Another limitation is that the cohort is not gender balanced; it consists of mainly male patients (72%). We planned to include all unselected consecutive patients referred to the stroke unit who underwent MRI, however, many patients with ICH could not undergo MRI for being too sick or with contraindications, which led to a potential for selection bias. All patients were randomly allocated to two different MRI scanners due to the availability, which might be a potential source of image heterogeneity. However, two scanners have the same field strengths and similar ICH scanning protocols that limit image heterogeneity influencing the ratings. We only rated the PVS in BG and CS regions, however, we did not rate the PVS in the midbrain because there were few numbers of slices on which midbrain PVS appear and its rating agreement was influenced by the limited slice number and partial volume effect. We did not include the hippocampus region because of its visualization on axial images varied and may be confused with normal variants hippocampal fissural cysts as suggested previously (52). However, we rated the BG and CS PVS using a validated scale widely used in stroke and SVD studies (10, 21) and we reviewed all available slices instead of a typical one slice in order to increase rating accuracy. Future validation and confirmation of these results in larger, prospective cohorts will be needed to surmount these sampling biases and current limitations.

The present study provides preliminary evidence for the risk factors and neuroimaging features for MR-visible PVS in spontaneous ICH patients, which are age, periventricular WMH, and presence of microbleeds in the BG region and alcohol-drinking habit, hypercholesterolemia history, and strictly lobar microbleeds in the CS region. These factors have potential applications for PVS in clinical research or clinical trials and may be an efficient way to explore the biological mechanisms underlying PVS in ICH patients. Further studies are needed to explore the mechanism and clinical importance of PVS, with possible implications for cerebrovascular disease prevention.

## Data Availability

All datasets generated for this study are included in the manuscript and/or the supplementary files.

## Ethics Statement

All 306 patients participating in this study provided written informed consent. The Beijing Tiantan Hospital Ethics Committee approved this study.

## Author Contributions

XW and XZ designed the study and drafted the manuscript. HF and YW collected the clinical data and managed the database. JZ collected the imaging data in this study. All authors approved for the manuscript submitted.

### Conflict of Interest Statement

The authors declare that the research was conducted in the absence of any commercial or financial relationships that could be construed as a potential conflict of interest.
